# Prediction of visual function from automatically quantified optical coherence tomography biomarkers in patients with geographic atrophy using machine learning

**DOI:** 10.1038/s41598-022-19413-z

**Published:** 2022-09-16

**Authors:** Konstantinos Balaskas, S. Glinton, T. D. L. Keenan, L. Faes, B. Liefers, G. Zhang, N. Pontikos, R. Struyven, S. K. Wagner, A. McKeown, P. J. Patel, P. A. Keane, D. J. Fu

**Affiliations:** 1 NIHR Biomedical Research Centre at Moorfields Eye Hospital NHS Foundation Trust, UCL Institute of Ophthalmology, Moorfields Reading Centre and Clinical AI Hub, 162 City Rd, London, EC1V 2PD UK; 2 Division of Epidemiology and Clinical Applications, National Eye Institute, National Institutes of Health, Bethesda, MD USA; 3 Apellis Pharmaceuticals, Inc, Waltham, MA USA; 4 Department of Ophthalmology, Erasmus University Medical Center, Rotterdam, The Netherlands

**Keywords:** Predictive markers, Software, Computer science, Medical imaging

## Abstract

Geographic atrophy (GA) is a vision-threatening manifestation of age-related macular degeneration (AMD), one of the leading causes of blindness globally. Objective, rapid, reliable, and scalable quantification of GA from optical coherence tomography (OCT) retinal scans is necessary for disease monitoring, prognostic research, and clinical endpoints for therapy development. Such automatically quantified biomarkers on OCT are likely to further elucidate structure–function correlation in GA and thus the pathophysiological mechanisms of disease development and progression. In this work, we aimed to predict visual function with machine-learning applied to automatically acquired quantitative imaging biomarkers in GA. A post-hoc analysis of data from a clinical trial and routine clinical care was conducted. A deep-learning automated segmentation model was applied on OCT scans from 476 eyes (325 patients) with GA. A separate machine learning prediction model (Random Forest) used the resultant quantitative OCT (qOCT) biomarkers to predict cross-sectional visual acuity under standard (VA) and low luminance (LLVA). The primary outcome was regression coefficient (r^2^) and mean absolute error (MAE) for cross-sectional VA and LLVA in Early Treatment Diabetic Retinopathy Study (ETDRS) letters. OCT parameters were predictive of VA (r^2^ 0.40 MAE 11.7 ETDRS letters) and LLVA (r^2^ 0.25 MAE 12.1). Normalised random forest feature importance, as a measure of the predictive value of the three constituent features of GA; retinal pigment epithelium (RPE)-loss, photoreceptor degeneration (PDR), hypertransmission and their locations, was reported both on voxel-level heatmaps and ETDRS-grid subfields. The foveal region (46.5%) and RPE-loss (31.1%) had greatest predictive importance for VA. For LLVA, however, non-foveal regions (74.5%) and PDR (38.9%) were most important. In conclusion, automated qOCT biomarkers demonstrate predictive significance for VA and LLVA in GA. LLVA is itself predictive of GA progression, implying that the predictive qOCT biomarkers provided by our model are also prognostic.

## Introduction

Geographic atrophy (GA) is a chronic progressive degeneration of the macula, the central 24 mm^2^ (20°) region of the retina required for central vision. It is the defining lesion of late age-related macular degeneration (AMD)^1,2^. GA is associated with significant, irreversible vision loss. To assess visual function in AMD, visual acuity (VA) measured using an eye chart under standardised lighting is an established key outcome measure as defined by the International Consortium for Health Outcomes Measurement^3^. Interestingly, in early stages of AMD, VA measured under low luminance conditions (low luminance visual acuity [LLVA]) can be reduced whilst standard VA remains unaffected^4,5^. Most patients initially present with non-central GA (affecting the parafoveal region of the macula) and gradually proceed to foveal involvement (central GA)^6–8^. In this context, LLVA correlates with future deterioration of VA and its reduction indicates that the patient will head towards the beginning of end-stage disease, and loss of foveal function may be impending. Yet the physiological mechanism underlying this outcome measure is unknown^9,10^.

With no current treatments for GA and promising therapies on the horizon^11–13^, it is increasingly important to establish structure–function relationships within GA, as they: (i) provide further understanding of the pathophysiological mechanisms underlying GA; (ii) refine monitoring of disease activity and thereby diagnostic and prognostic counselling at the individual patient level; (iii) serve to define clinical endpoints in clinical trials and enable identification of earlier stages, i.e. opportunities for interventions that can prevent vision loss.

The current reference standard for diagnosing, characterising, and monitoring progression in GA is spectral domain optical coherence tomography (SD-OCT), as it captures the cross-sectional morphology of retinal structures^14^. Indeed, an international consortium of experts in AMD and retinal imaging—the Consensus of Atrophy Meetings (CAM) group—chose to define disease progression in GA based on SD-OCT structural markers^1,15^). Their proposed terms for macular atrophy in the context of AMD each describe the affected anatomical layers and represent distinct disease stages. Herein, complete RPE (retinal pigment epithelium) and outer retinal atrophy (cRORA) represents the endpoint of atrophy—encompassing GA—and is defined by regions of: choroidal hypertransmission with diameter ≥ 250 µm; RPE attenuation or disruption with diameter ≥ 250 µm; overlying photoreceptor degeneration; and absence of RPE tear^15^. Regions in which these features overlap but are less than 250 µm are termed incomplete RPE and outer retinal atrophy (iRORA)—a precursor stage to cRORA^16^. Recently, we developed a deep learning model to automatically segment RPE-loss, photoreceptor degeneration, and hypertransmission from OCT scans and was shown through external validation to be comparable with human specialist efforts^17^. Regions in which these features overlap represent RORA (RPE and outer retinal atrophy) and can be considered as a continuous variable that encompasses both cRORA (GA) and iRORA.

To date, the relationship between OCT structural features and visual function in GA has relied on manual segmentation and does not consider the biomarkers defined in the CAM consensus statement^18,19^. This study makes use of an externally-validated algorithm that automatically segments these quantitative OCT (qOCT) biomarkers, applying it to non-neovascular AMD datasets with GA (n = 476) from both a clinical trial and routine clinical care. Applying machine learning modelling, both standard and low luminance VA could be predicted to a level that has yet to be achieved. Furthermore, spatial localisation and severity of anatomical disruption of qOCT biomarkers were mapped onto the macula providing further insight into structure–function relationship in GA and the (otherwise unknown) underlying physiological mechanism of early LLVA impairment.

## Methods

### Study design

This is a non-interventional, post hoc analysis of patients with GA secondary to non-neovascular AMD. Reporting adhered to guidelines for observational studies put forth by the Strengthening the Reporting of Observational Studies in Epidemiology (STROBE) statement^20^.

### Study cohort

This study considered data from two sources: participants enrolled in the FILLY trial (NCT02503332)^11,21^ and real-world data collected as part of routine clinical care for patients with GA at Moorfields Eye Hospital NHS Foundation Trust, London, United Kingdom.

The FILLY trial was a phase II, international, multicenter clinical trial assessing safety, tolerability, and evidence of activity of intravitreal pegcetacoplan in eyes with GA secondary to non-neovascular AMD with best-corrected visual acuity (BCVA) greater than 24 Early Treatment Diabetic Retinopathy Study (ETDRS) letters (Supplementary Methods 1 and Supplementary Fig. 1). Here, only baseline trial data were considered, i.e. the timepoint prior to initiation of the intervention. The VA values represent BCVA testing using ETDRS charts by certified examiners after refraction. BCVA under low luminance conditions (low luminance visual acuity; LLVA) was measured as for BCVA but with a 2.0 log neutral density filter covering the eye. The study eye was assessed first, after allowing for adequate time for adaptation to low luminance conditions, empirically determined. LLVA was measured prior to BCVA to avoid memorisation of letters. Low luminance deficit (LLD) was defined as the difference between BCVA and LLVA (i.e. BCVA–LLVA).

Patients from Moorfields Eye Hospital were included if all of the following criteria were met (Supplementary Fig. 1): they attended a medical retina clinic between 01-January-2016 and 31-January-2019; GA was included as a term in the clinic correspondence letter; Heidelberg OCT scans (greater than 25 b-scans per volume) were obtained from both eyes within 15 days of the appointment; absence of prior anti-VEGF therapy; and the OCT scans were confirmed to contain GA secondary to non-neovascular AMD (according to manual validation by a Reading Centre expert grader at the Moorfields Reading Centre). Here, VA (with habitual correction or pinhole) was measured as part of a clinical examination with an ETDRS chart. Pinhole VA was used if better than VA with habitual correction. For any eyes with multiple timepoints meeting the eligibility criteria, the earliest timepoint was selected.

For all patients, only a single macular OCT scan and a corresponding VA measurement were considered per eye. The luminance range of ETDRS charts across the two patient cohorts was 85–120 cd/m^2^. All OCT volumes were acquired using Heidelberg Spectralis OCT (Heidelberg Engineering, Heidelberg, Germany) having equal to or greater than 25 b-scans covering 6 × 6 × 2 mm^3^.

The study was conducted in compliance with the tenets of the Declaration of Helsinki and has approval from the Moorfields Eye Hospital Institutional Review Board (research reference: ROAD17/031, clinical audit reference: CA17/MR/28). The requirement for informed consent was waived by the national health research and ethics review board of England (Health Research Authority of England, Project ID: 281957, Protocol number: KEAP1006, Research Ethics Committee Reference: 20/HRA/2158) as this is the standard for use of retrospective, de-identified data for research within the UK National Health Service (NHS).

### Image analysis workflow

All OCT volumes were processed using validated deep learning models^17^. The deep-learning models were developed as a variation of the U-Net architecture. Briefly, for every pixel of an input image, the model outputs a likelihood estimate for a given feature. Models were trained for each of the morphological features that define geographic atrophy: RPE loss, overlying photoreceptor degeneration, and hypertransmission. A fourth model that segments RORA was also trained. RORA was defined as overlapping regions of RPE-loss, photoreceptor degeneration, and hypertransmission, i.e. any retinal areas with all three features present and therefore encompassing both iRORA and cRORA^15,22^. The models were applied on every OCT volume scan and performed automated segmentation of each of the 49 b-scans per volume.

**Figure 1:**
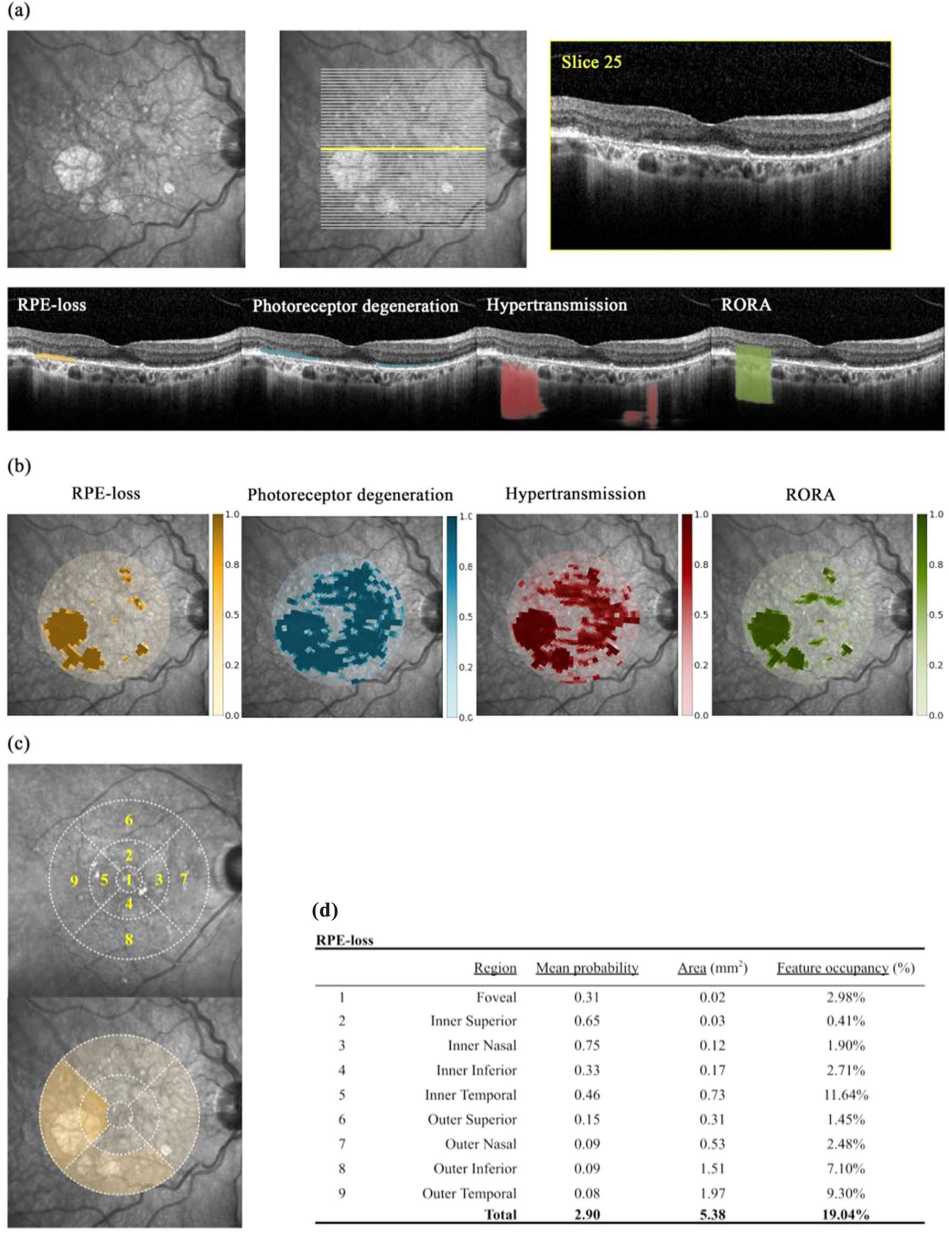
Image analysis workflow. (**a**) For each OCT volume, all b-scans were segmented for RPE-loss (orange), photoreceptor degeneration (blue), hypertransmission (red), and RPE and outer retinal atrophy (RORA; green). RORA is taken to be overlapping regions of the three former features i.e. co-occurrence as per a-scan. Exemplar segmentation of a single b-scan and its axis along en face fundus photograph. (**b**) Resultant feature probability maps from total volume segmentations collectively presented by projection onto en face fundus photograph. Colour legends represent target feature probability. Manual central foveal point annotation permitted interpolation of a given voxel’s localisation in relation to the fovea. (**c**) ETDRS regions were also considered wherein the macula is considered as a 6 mm diameter circle divided into 9 areas: central foveal area (1 mm diameter); 4 parafoveal (collectively span 3 mm diameter); and 4 perifoveal areas. (**d**) Here, the mean feature probability within each region is displayed.

For each two-dimensional b-scan, the outputs of the models on a pixel level were converted into a one-dimensional binary label, representing the presence or absence of the feature per vertical column (A-scan). (Fig. 1a)^17^.

The automatic segmentation process assigns a probability for each of the features to each voxel within an OCT volume. Voxel spatial localisation was interpolated in relation to the central fovea, thereby standardising locations and enabling comparison between patients (Fig. 1b). Central foveal points were manually annotated by a reading centre expert grader at the Moorfields Reading Centre. The spatial localisation of feature probabilities was also considered by dividing the macula into each of the ETDRS regions, i.e. mean probability within each of the nine ETDRS regions (foveal, 4 parafoveal, and 4 perifoveal areas for the nasal, temporal, superior, and inferior regions) (Fig. 1c). The area of each segmented feature in square millimeters (mm^2^) was considered by applying optimised probability thresholds identified from the original model development and validation (Fig. 1d)^17^.

### Predicting visual acuity

A random forest regression model was trained using the segmentation output (i.e. the raw probabilities at the voxel level for each feature (RPE-loss, photoreceptor degeneration, hypertransmission, and RORA) as input variables to predict cross-sectional VA in ETDRS letters. Three individual instances of the same model were trained: one with OCTs from the clinical trial FILLY cohort, one with OCTs from the MEH cohort, and one with OCTs from a combined Overall cohort. Regarding prediction of VA from qOCT biomarkers under standard-luminance conditions, the three models were evaluated for: (i) VA under RCT conditions i.e., FILLY; (ii) VA from real-world routine care, i.e., MEH; and (iii) VA from the Overall cohort.

In separate analyses, VA under low luminance conditions and Low Luminance Deficit (LLD), only available in the FILLY study cohort, were each considered as the dependent variable for separate instances of the random forest regression model.

The goodness of model fit was evaluated by comparing the regression coefficient (r^2^) and mean absolute error (MAE) computed from 100-fold bootstrapped random forest models trained on 80% of the bootstrap sample and evaluated on the other 20% split at the patient level. Feature importance was calculated by the method incorporated in the scikit-learn implementation of random forest regression, whereby features’ rankings for their variance reduction capability are averaged over the ensemble. Output was multiplied by 100 to give percentage contribution towards model performance. Analyses were carried out with Python (version 3.6.9) and summarised metrics in the text are expressed as median with ± interquartile range (IQR), unless otherwise specified, as the measurements were not normally distributed.

### Meeting presentation

EURETINA 2021 Prize Paper Presentation—Artificial Intelligence session.

## Results

### Cohort demographics

The study cohort comprised 476 eyes with GA secondary to non-neovascular AMD from 325 patients that were undergoing a clinical trial of pegcetacoplan in GA secondary to AMD (n = 195) or routine clinical care at a large UK tertiary centre (n = 130) (Supplementary Fig. 1). Mean (SD) age was similar across groups, with 80.1 (7.6) and 79.1 (9.1) years in the FILLY and MEH cohorts, respectively. Overall median age was 80.5 ± IQR 11.1 years. In both cohorts, the majority were female (FILLY: 63.1%; MEH: 56.2%) (Table 1). No patient had a history of treatment with hydroxychloroquine or chloroquine.

**Table 1:** Cohort demographics. Mean, median, minimum value, maximum value, standard deviation (SD), and proportional distributions are shown for gender, ethnicity, and age of cohorts including patients with geographic atrophy secondary to non-neovascular AMD. Patients were recruited as part of an international, multicentre trial (FILLY) or retrospectively through real-world clinical care at Moorfields Eye Hospital (MEH). Direct comparison of ethnicity was infeasible as each cohort represented ethnicities with distinct categorical variables that could not be intuitively merged.

	Overall (n = 325)	FILLY (n = 195)	MEH (n = 130)
**Gender**			
Female	196 (60.3%)	123 (63.1%)	73 (56.2%)
Male	129 (39.7%)	72 (36.9%)	57 (43.8%)
**Age (years)**			
Mean (SD)	79.7 (8.21)	80.1 (7.55)	79.1 (9.12)
Median [Min, Max]	80.5 [45.9, 103]	80.7 [60.4, 97.2]	79.8 [45.9, 103]
**Ethnicity**			
Afro Caribbean	–	1 (0.5%)	1 (0.8%)
Asian	–	–	6 (4.6%)
Caucasian	–	187 (95.9%)	62 (47.7%)
Other	–	2 (1.0%)	0 (0%)
Unknown	–	0 (0%)	61 (46.9%)
Hispanic or Latino	–	5 (2.6%)	–

### Clinical features and GA segmentation

A wide distribution in standard VA was observed in both study cohorts: median standard BCVA was 57.5 ± IQR 30.0 in the FILLY cohort and median best recorded VA was 50.9 ± 35.0 ETDRS letters in the MEH cohort, giving an overall median standard VA 60.5 ± IQR 32.0 ETDRS letters. Similarly, the other visual function metrics available for the FILLY cohort were widely distributed. LLVA was 32.0 ± 28.0, and LLD 21.0 ± 21.0 (Table 2 and Supplementary Fig. 2a).

**Table 2:** Visual function and corresponding GA feature segmentation. Mean, standard deviation (SD), and distribution are shown for (a) visual function metrics (standard visual acuity [VA] in ETDRS [early treatment diabetic retinopathy study] letters], low-luminance visual acuity [LLVA], low-luminance deficit [LLD, difference between VA and LLVA]); (b) feature segmentations (total areas in square millimeters [mm^2^] of RPE-loss, photoreceptor degeneration, hypertransmission, and geographic atrophy; (c) proportion of each feature overlapping with central foveal region (0.79 mm^2^). Cohort data was collectively summarised (Overall) and sub-stratified by recruitment (FILLY trial or MEH [Moorfields Eye Hospital]). LLVA and LLD were not measured as part of routine clinical care at MEH and these metrics were not available for 2 persons of the FILLY sub-cohort—these were collectively referred to as missing.

(a)	Overall (N = 476)	FILLY (N = 299)	MEH (N = 177)
Patients	325	195	130
**Standard visual acuity (ETDRS letters)**			
Mean (SD)	55.1 (21.1)	57.5 (18.5)	51.2 (24.4)
Median [Min, Max]	60.5 [0, 90.0]	60.0 [0, 90.0]	61.0 [0, 89.0]
**Low-luminance visual acuity**			
Mean (SD)	33.3 (17.5)	33.3 (17.5)	–
Median [Min, Max]	32.0 [0, 80.0]	32.0 [0, 80.0]	–
Missing	179 (37.6%)	2 (0.7%)	177 (100%)
**Low-luminance deficit**			
Mean (SD)	24.0 (16.2)	24.0 (16.2)	–
Median [Min, Max]	21.0 [0.00, 73.0]	21.0 [0.00, 73.0]	–
Missing	179 (37.6%)	2 (0.7%)	177 (100%)

(b)	Overall (N = 476)	FILLY (N = 299)	MEH (N = 177)
Patients	325	195	130
**RPE-loss (mm**^**2**^**)**			
Mean (SD)	8.53 (5.18)	8.21 (4.29)	9.06 (6.37)
Median [Min, Max]	7.82 [0.0309, 25.0]	7.49 [0.603, 20.8]	8.17 [0.0309, 25.0]
**Photoreceptor degeneration (mm**^**2**^**)**			
Mean (SD)	14.2 (6.38)	13.8 (5.60)	14.7 (7.49)
Median [Min, Max]	14.4 [0.493, 28.1]	13.4 [3.12, 28.1]	16.0 [0.493, 28.0]
**Hypertransmission (mm**^**2**^**)**			
Mean (SD)	9.70 (5.36)	9.44 (4.50)	10.2 (6.56)
Median [Min, Max]	9.23 [0.227, 24.8]	8.79 [1.23, 22.0]	9.59 [0.227, 24.8]
**RORA (mm**^**2**^**)**			
Mean (SD)	7.24 (4.77)	6.91 (3.87)	7.79 (5.97)
Median [Min, Max]	6.22 [0.0806, 24.0]	6.03 [0.584, 17.5]	6.56 [0.0806, 24.0]

(c)	Overall (N = 476)	FILLY (N = 299)	MEH (N = 177)
Patients	325	195	130
**RPE-loss (%)**			
Mean (SD)	61.1 (38.2)	62.4 (38.2)	58.9 (38.2)
Median [Min, Max]	77.0 [0, 99.0]	80.0 [0, 99.0]	73.0 [0, 99.0]
**Photoreceptor degeneration (%)**			
Mean (SD)	85.5 (26.5)	86.7 (25.8)	83.5 (27.6)
Median [Min, Max]	99.0 [0, 99.0]	99.0 [0, 99.0]	99.0 [0, 99.0]
**Hypertransmission (%)**			
Mean (SD)	67.5 (34.8)	69.0 (34.0)	65.0 (36.0)
Median [Min, Max]	83.0 [0, 99.0]	84.0 [0, 99.0]	80.0 [0, 99.0]
**RORA (%)**			
Mean (SD)	53.6 (38.4)	54.3 (38.8)	52.5 (37.7)
Median [Min, Max]	62.0 [0, 99.0]	62.0 [0, 99.0]	60.0 [0, 99.0]

Automatic segmentation of the Overall cohort’s OCTs revealed that across all parameters analysed, the MEH cohort featured on average larger areas affected by GA (Table 2 and Supplementary Fig. 2b). Median (IQR) RORA was 6.03 mm2 (5.59) in the FILLY cohort versus 6.6 (9.32) in the MEH cohort. Similarly, mean areas affected by RPE loss, photoreceptor degeneration and hypertransmission were nominally larger in the MEH cohort compared to the FILLY cohort. The total area occupied by each feature was highly variable in the Overall cohort, resulting in a distribution with median values of: 7.82 ± 7.58 mm^2^ RPE-loss, 14.4 ± 9.57 mm^2^ photoreceptor degeneration, 9.23 ± 7.58 mm^2^ hypertransmission, and 6.22 ± 6.49 mm^2^ RORA (Table 2 and Supplementary Fig. 2b). Presence of RORA and its constituent features was also highly variable within each region, ranging from completely absent (0%) to confluent (> 99%). Indeed, RORA was wholly absent from the circular ETDRS foveal region (diameter 1 mm; 0.79 mm^2^) in 14.7% of all OCT volumes (70/476; 41/299 FILLY and 29/177 MEH) (Table 2).

### Prediction of standard visual acuity using machine learning

**Figure 2:**
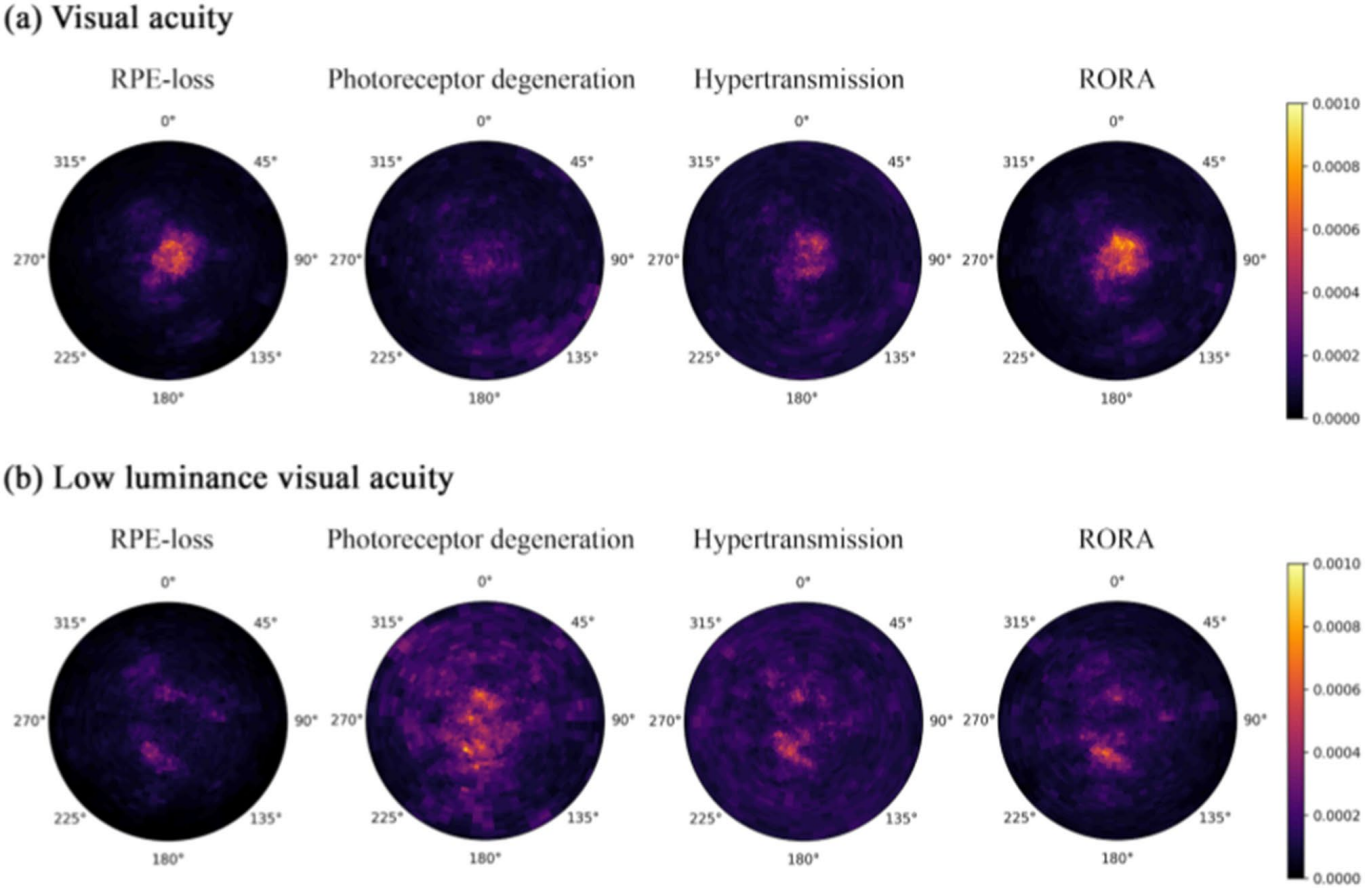
Heatmap of relative feature predictive value. Normalised random forest feature importance as a measure of the predictive value of the four considered features of GA; RPE-loss, photoreceptor degeneration, and hypertransmission and RORA and their locations relative to the fovea to the predicted value for (**a**) standard visual acuity (Overall cohort) and (**b**) low luminance visual acuity (FILLY cohort). Feature importance values were averaged across 100 bootstraps of the dataset.

For standard VA, the accuracy of using automatically segmented feature probabilities at the voxel level to predict VA was higher in the FILLY cohort (r^2^ 0.46 MAE 10.2) than the real-life MEH cohort (r^2^ 0.30 MAE 15.3). The random forest regression model trained on the two combined cohorts (Overall cohort) had r^2^ 0.40, with mean absolute error (MAE) of 11.7 ETDRS letters (Table 3a). Normalised random forest feature importance, as a measure of the predictive value of the three constituent features of GA; RPE-loss, PDR, hypertransmission and their locations, was reported both on voxel-level heatmaps (Fig. 2a) and ETDRS-grid subfields (Table 3b). Predictive importance for each feature and its position was ranked, wherein RORA contributed the most (30.5% in Overall cohort; 30.9% in FILLY and 28% in MEH), followed by hypertransmission (25.1% Overall; 25.7% FILLY 26.8% MEH), RPE-loss (23.7% Overall; 24.8% FILLY 22.4% MEH), and photoreceptor degeneration (20.7% Overall; 18.8% FILLY 22.9% MEH) (Fig. 2a and Table 3b). Feature ranking was further considered by summing feature importance across ETDRS regions. Critically, features within the foveal region contributed the most (46.5% Overall; 47.1% FILLY; 46.5% MEH) despite being the smallest ETDRS region—0.78 mm^2^ (Table 3b).

**Table 3:** Structure–function correlation between qOCT biomarkers of GA area and VA. (a) A random forest regression model was trained using the deep-learning segmentation output (i.e. the raw probabilities at the voxel level for each feature (RPE-loss, photoreceptor degeneration, hypertransmission, and RORA) as input variables to predict cross-sectional VA under standard luminance conditions, low-luminance VA, and low-luminance deficit in ETDRS letters. For VA under standard-luminance conditions, separate models were evaluated for: (i) BCVA under RCT conditions i.e., FILLY; (ii) VA from real-world routine care, i.e., MEH; and (iii) a third that combines the two. Model bootstrapped 100-fold with resultant regression coefficients (r^2^) and mean absolute error (MAE) shown. Importance of qOCT biomarker features in predicting (b) standard visual acuity and (c) low luminance visual acuity was queried using machine learning. Random forests modelling was used to evaluate value of the qOCT biomarkers RPE-loss, photoreceptor degeneration, hypertransmission, and RORA in predicting cross-sectional visual acuity under standard lighting conditions (Overall model). The resultant adjusted feature importance values were summed according to location within ETDRS region and multiplied by 100 to give the percentage contribution towards the model's performance. For example, RORA within the foveal region accounted for 16.8% of the model’s performance of r^2^ 0.40 MAE 11.7 ETDRS letters for standard visual acuity.

(a)	R^2^	MAE (25% quartile—75% quartile)				
**Standard visual acuity**						
Overall	0.40	11.7 (11.2–12.4)				
FILLY	0.46	10.2 (9.5–10.7)				
MEH	0.30	15.3 (13.8–16.6)				
**Low-luminance visual acuity**						
FILLY	0.25	12.1 (11.4–12.9)				
**Low-luminance deficit**						
FILLY	0.25	10.1 (9.5–10.8)				

Region		RPE-loss	Photoreceptor degeneration	Hypertransmission	RORA	Sum
**(b) Standard visual acuity**						
1. Foveal		13.4	6.4	8.5	14.8	43.1
2. Inner superior		2.3	1.6	2.5	3.1	9.5
3. Inner nasal		1.7	1.7	2.0	2.6	8.9
4. Inner inferior		1.7	1.6	1.9	2.1	7.3
5. Inner temporal		1.5	1.8	1.7	2	7.0
6. Outer superior		0.8	1.8	2.1	1.4	6.1
7. Outer nasal		0.9	2.1	2.3	1.7	7.0
8. Outer inferior		0.7	2.3	2.4	1.6	7.0
9. Outer tempora		0.6	1.4	1.8	1.2	5.0
Sum		23.7	20.7	25.1	30.5	

Region		RPE-loss	Photoreceptor degeneration	Hypertransmission	RORA	Sum
**(c) Low luminance visual acuity**						
1. Foveal		3.0	12.2	5.6	4.8	25.5
2. Inner superior		2.8	4.0	2.9	3.0	12.7
3. Inner nasal		1.7	3.1	2.2	2.2	9.2
4. Inner inferior		1.9	4.1	2.7	2.9	11.6
5. Inner temporal		1.6	3.3	2.3	2.4	9.5
6. Outer superior		0.9	3.7	2.7	1.7	8.9
7. Outer nasal		0.9	3.0	2.5	1.7	8.1
8. Outer inferior		0.5	3.0	2.5	1.3	7.3
9. Outer temporal		0.7	2.5	2.5	1.5	7.1
Sum		13.8	38.9	26.0	21.4

### Prediction of low luminance visual acuity using machine learning

Additional visual parameters were available in the FILLY cohort. Here, random forest cross-sectional predictions of LLVA and LLD from Deep-Learning segmentation model output feature probabilities. Regression coefficients (r^2^) of 0.25 (MAE 12.1) and 0.25 (MAE 10.1) were observed for LLVA and LLD, respectively (Table 3a). Ranking of feature importance for LLVA as visualised in corresponding heatmaps was led by photoreceptor degeneration (38.9%), followed by hypertransmission (26.0%), RORA (21.4%), and RPE-loss (13.8%) (Fig. 2b and Table 3c). In contrast to standard VA, where feature importance was greatest for the foveal region, features at non-foveal regions were most important in predicting LLVA. When the VA and LLVA random forest regression models were repeated with one eye per patient, a similar pattern of feature importance was observed (Supplementary Table 1). The correlation between actual and predicted values for VA and LLVA and the corresponding Bland-Altman plots are presented in Supplementary Figure 3.

## Discussion

### Main findings

Our data demonstrate that both standard and low luminance VA in patients with GA secondary to non-neovascular AMD can be predicted using qOCT features that have been automatically segmented and quantified. This is the first time a structure–function relationship has been described for LLVA, creating a tool to assess its underlying physiological mechanisms. The cross-sectional predictive performance demonstrated for standard VA in the Overall cohort (r^2^ = 0.40; Table 3a) is superior to that from similar efforts in neovascular AMD also using machine-learning based segmentation algorithms—r^2^ = 0.11–0.21^23,24^. An MAE of 11.7 ETDRS letters is a step towards the limit of predicting VA, as the test–retest repeatability for standard VA is 5–6 ETDRS letters in eyes without disease and thought to be even greater in eyes with GA^25,26^.

### Mapping structure and location using probability heatmaps

The algorithm described here can produce a “GA feature probability heatmap”, wherein the algorithm output of segmentation probabilities is interpolated in relation to the fovea and projected onto an en face fundus image. Probability maps allow us to instantly consider quantitative segmentation data across an entire image volume simultaneously through an intuitive graphic. Consideration of segmentation features as continuous variables (i.e. probability of feature presence) rather than binary variables (feature present or not present) obviates difficulties of assigning fixed thresholds. Landmarking each voxel to a reference point enables comparison of features over time and even between samples and patients (an obligate step for modelling).

### Structure–function correlation of standard visual acuity

A stratified feature-region analysis based on the Overall cohort suggested that foveal RORA, followed by foveal RPE-loss, is the strongest predictor of standard VA in GA. The strong foveal contribution to predicting standard VA is largely unsurprising, reflecting the clinical observation that central GA is accompanied by poor VA^7^. Indeed, topographical analyses of fundus autofluorescence as well as SD-OCT have demonstrated that foveal sparing is an independent covariate of GA^27^ and that VA is likely to be worse in eyes with definitive foveal involvement^28^. To date, a correlation between total GA area and VA has not been readily apparent using fundus photography^27–31^. Here, we present evidence to show SD-OCT imaging can also detect correlations between non-foveal features and VA. This may be because fundus photography only provides two-dimensional, en face representations of the retina, limiting in-depth assessment of the retinal layers and discrimination between histological subtypes of GA and their respective contribution to functional deficit^32^. Macular segmentation with SD-OCT thus presents a potentially more sensitive modality for GA and its sequelae on visual function.

Currently, GA is usually considered present when the lateral spread of RORA affects an area of atrophy ≥ 250 μm in diameter^15^. Using the algorithm described here allows evaluation of RORA as a continuous variable, and thresholding based on the extent of the lateral spread may be applied as a secondary step. This may facilitate the continuous monitoring and evaluation of GA in a research context, with a view to eventually develop clinical monitoring and preventative strategies.

### Predicting standard visual acuity

Separate instances of the ML prediction model for standard VA were trained on two datasets: one originating from a Randomised Clinical Trial (the FILLY study) and one from real-life clinical practice at Moorfields Eye Hospital. The resulting regression coefficients showed a higher predictive performance in the RCT than the real-life cohort, which wasn’t unexpected. There are differences in the protocol for VA measurement between the two cohorts and VA data from real-life clinical practice is likely to contain higher levels of noise. These contributing factors play certainly a role in explaining the observed discrepancy. It is worth noting, however, that VA measurements at Moorfields Eye Hospital follow a standardised protocol delivered by trained staff with high levels of adherence and the inter-session repeatability for standard VA from MEH cohorts previously reported was comparable to that encountered in clinical trial settings^26^. The smaller sample size of the MEH cohort may also have contributed to this discrepancy as increased diversity and heterogeneity within larger training datasets improve predictive performance of Random Forest prediction models.

Despite the difference in strength of correlation, the fact that Feature Importance ranking in the two cohorts with ML methods led to the identification of very similar anatomical features (RPE loss, RORA) and geographical location (foveal area) on OCT scans as more predictive of standard visual acuity in both cohorts, is novel and provides new insight into the pathophysiology of GA, especially in conjunction with the Feature Importance findings for LLVA reported below.

A third instance of the ML prediction model for standard VA was trained on combined datasets of the two cohorts and showed overall predictive performance higher than previously reported in relevant literature. It also confirmed Feature Importance ranking of individual cohorts, as depicted in the corresponding probability heatmap (Fig. 2a). The Overall prediction model is likely to produce more accurate predictions than the two separately trained models given the properties of the Random Forest ML methodology used in this study.

Random Forests are an ensemble learning method for both classification and regression. Ensemble learning models in ML yield better results when there is diversity among the models they combine. Random Forests apply bootstrapping to decrease *variance* of resultant models (thus preventing *overfitting*), without increasing *bias* (thus preventing *underfitting*)^33^. This means that increasing data diversity leads to decreased sensitivity to noise and improved prediction accuracy. The Random Forest model developed on the combined datasets from an RCT and a real-life clinical practice cohort is thus more likely to generalise to other patient cohorts predicting VA values that are closer to the ‘true’ VA in each case. A further property of Random Forest modeling, known as ‘feature bagging’, involves random subset feature selection at each split, thus achieving the de-correlation of features in the training set^34^. This ensures that Feature Importance ranking is a true representation of each feature’s independent contribution to the prediction, while preventing the inflation of importance of genuinely strong predictors, which could be caused by strong correlation among features. In training our models, we performed 100-fold bootstrap resampling.

### Predicting low luminance visual acuity

LLVA is a simple, inexpensive, quick assessment with standard ophthalmic equipment and has a test–retest repeatability (between 1.6 and 1.9 logMAR [5–6.5 ETDRS letters]) comparable to standard luminance VA^35,36^. It has been shown to correlate with microperimetry retinal sensitivities^37^ and patient-reported night vision symptoms^38^. LLVA is also an earlier clinical marker of change in central retinal function than standard VA. LLVA deterioration precedes deterioration in standard VA and thus predicts impending loss of foveal function. This is consistent with the structure–function correlation analyses presented here, as the most predictive features differed between the two measures of visual function: photoreceptor degeneration for LLVA and RPE-loss and RORA for standard VA. This aligns with previous observations that photoreceptor degeneration can precede RPE-loss and eventual RORA in GA, as well as with current understanding that RPE dysfunction is common to all, or at least most, early AMD^39,40^. That is, photoreceptor cells are metabolically dependent on RPE and therefore degeneration arises secondarily to RPE dysfunction, which itself eventually atrophies. Furthermore, features within non-foveal areas were more predictive of LLVA than for standard VA. This may reflect the very high density of cones within the foveola vs. the para-fovea. Hence redundancy in the foveola may ostensibly mask early standard luminance vision loss, yet low luminance visual acuity is affected earlier coinciding with photoreceptor degeneration in the para-foveal area due to lack of redundancy.

This might also support the hypothesis that low light sensitivity is mediated by a circuit function of horizontal and amacrine cells within the plexiform layers and thus a larger area of preserved central macula is required for LLVA^41^.

### Limitations

VA is the most commonly used functional measure to evaluate the visual system. It is widely accepted in clinics and by regulatory authorities as a key measure of visual function and represents the gold standard by which the efficacy of treatment is judged. It correlates with quality of life and defines key functional thresholds, such as eligibility for driving and for sight impairment registration. However, VA change over time is non-linear, can improve from one timepoint to the next, and does not wholly capture the sequelae of GA on visual function, as it largely represents central acuity of the fovea^42^. Other functional manifestations include parafoveal functions such as dark adaptation, reading speed, face recognition, and perimetry. Foveal-sparing disease (i.e. not affecting VA) can impact other visual functions (including reading speed, contrast sensitivity, fixation, and VFQ-25)^43^. Changes in visual function can even occur prior to VA deterioration. Thus, other markers of vision-related performance in everyday life should be considered to complement VA in patients with GA. This study only considers cross-sectional VA values, wherein future enquiry would benefit from consideration of future VA and whether that can be predicted.

### Conclusion

Our results demonstrate the utility of automatically segmented imaging biomarkers in predicting visual function. This is an important step towards standardising care by reliably predicting ‘true’ visual function from refined imaging biomarkers enabled by AI and may contribute to the development of point-of-care decision-aid systems for personalised ophthalmology. Here we have used this tool to further our insight into the (otherwise unknown) underlying physiological mechanism of LLVA and thereby progression from Intermediate AMD to GA and its subtypes.

## Supplementary Information

Supplementary Information.

Supplementary Table 1.

Supplementary Figure 1.

Supplementary Figure 2.

Supplementary Figure 3.

## Data Availability

FILLY Study Data: The FILLY study dataset, which was used under license for the current study, is not publicly available due to licensing restrictions. Moorfields Data: Mooorfields data analysed during the current study is not publicly available at present due to information governance policies pertaining to real-life clinical data. The de-identified Moorfields dataset will become available to the scientific community within the Data Governance framework of the National HDR UK INSIGHT Hub (https://www.insight.hdrhub.org/). Please address inquiries for updates and data access requests to the corresponding author (k.balaskas@nhs.net).
