# Supplementary material for: Prediction of visual function from automatically quantified optical coherence tomography biomarkers in patients with geographic atrophy using machine learning
Source: Sci Rep. 2022 Sep 16;12:15565. doi: 10.1038/s41598-022-19413-z (PMC9481631; doi:10.1038/s41598-022-19413-z)
Supplement: Supplementary file 1 — Supplementary Information. [file 41598_2022_19413_MOESM1_ESM.docx]

**SUPPLEMENTARY MATERIALS**

**Title: Machine-learning and automatically segmented retinal biomarkers generate spatial heatmaps predictive for standard and low luminance visual acuity in geographic atrophy**

**Index:**

Supplementary Methods 1: Page 2

Supplementary Figure Legend 1: Page 3

Supplementary Figure Legend 2: Page 3

Supplementary Figure Legend 3: Page 4

Supplementary Table Legend 1: Page 4

**Supplementary Methods 1:**

**FILLY trial IRB/Ethics Committee approvals and Patient Characteristics.**

The FILLY trial is a 18-month prospective, multicenter, randomised, sham-controlled phase 2 study that enrolled patients at 46 sites in the United States (New England Institutional Review Board, University of Miami, Mayo Clinic, Institutional Review Board of the Cleveland Clinic Foundation, Duke University Health System Institutional Review Board, and Research Compliance Office, Stanford University), Australia (Bellberry Ltd), and New Zealand (Northern A Health and Disability Ethics Committee, Health and Disability Ethics Committees, and Ministry of Health). The study was performed in accordance with the tenets of the Declaration of Helsinki, International Conference on Harmonization Good Clinical Practice guidelines, and all applicable regulations. Institutional review board or ethics committee approval was obtained at each site. All patients provided written informed consent.

Patients were included if equal or greater than 50 years of age and fulfilled the following major criteria with reference to the study eye: (i) best corrected visual acuity of 24 Early Treatment Diabetic Retinopathy Study letters (20/320 Snellen equivalent); (ii) diagnosis of GA secondary to AMD confirmed using fundus autofluorescence imaging with GA area size of 2.5 mm^2^ or more and 17.5 mm^2^ or less; (iii) presence of any pattern of hyper-autofluorescence in the junctional zone of GA; (iv) and at least 1 focal lesion of 1.25 mm^2^ or more if GA was multifocal. Geographic atrophy, exudative AMD, or both were permitted in the contralateral eye. An independent central reading center (Digital Angiography Reading Center, Great Neck, NY) confirmed lesion eligibility. Major exclusion criteria with reference to the study eye included GA secondary to causes other than AMD, history or current evidence of exudative AMD, and retinal disease other than AMD.

**Supplementary Figure and Table Legends**

**Supplementary Figure 1:**

**Cohort selection flow diagram**.

For the FILLY cohort (left panel), both study and fellow eyes of all patients enrolled in the FILLY trial (NCT02503332) were considered. Eyes with Heidelberg OCT scans greater than 25 b-scans per volume and those with GA secondary to non-neovascular AMD were taken forward in this study. For the Moorfields Eye Hospital (MEH) cohort (right panel), correspondence letters for patients older than 45 years of age attending medical retina clinic between 01-January-2016 and 31-January-2019 were screened for terms pertaining to a diagnosis of geography atrophy. Herein, patients were only considered if Heidelberg OCT scans (greater than 25 b-scans per volume) were obtained from both eyes within 15 days of the appointment and with absence of prior anti-VEGF therapy. Eyes were confirmed to have GA secondary to non-neovascular AMD by manual validation by a reading centre expert grader at the Moorfields Reading Centre and therefore a pragmatic, random sample was selected.

**Supplementary Figure 2:**

**Visual function and qOCT parameters at baseline**.

Depicted are box plots and vertical histogram of (**a**) standard visual acuity (VA; white), low-luminance visual acuity (LLVA; gray), and low-luminance deficit (difference between VA and LLVA; black) all in early treatment diabetic retinopathy study [ETDRS] letters). (**b**) Automatically segmented areas (mm^2^) of retinal pigment epithelium (RPE)-loss (yellow), photoreceptor degeneration (blue), hypertransmission (red), and RPE and outer retinal atrophy (RORA; green) are similarly displayed. Cohort data was collectively summarised (Overall) and sub-stratified by recruitment (FILLY or MEH).

**Supplementary Figure 3:**

**Predicted versus Observed Visual Acuity and Low-Luminance Visual Acuity**

1. **Plots of predicted versus observed values**

Horizontal axis: Observed values, Vertical axis: Predicted values (in ETDRS letters)

**Standard Visual Acuity:** Green Plot: FILLY study Best Corrected Visual Acuity values (BCVA), Red Plot: Moorfields Eye Hospital Visual Acuity values (VA)

**Low-Luminance Visual Acuity:** Blue Plot: FILLY study Low-Luminance Visual Acuity values (LLVA)

1. **Bland-Altman plots: Mean (blue line) and 95% Confidence Intervals (red lines)**

Horizontal axis: Mean values (in ETDRS letters), Vertical axis: Difference (Predicted minus Observed values)

Green, Red and Blue Plots corresponding to FILLY study VA, Moorfields Eye Hospital VA and FILLY study LLVA values respectively

**Supplementary Table 1:**

**Feature importance of structure-function correlations at patient-level split.**

From our cohort, one eye (randomly selected) per patient was taken forward and random forest regression models were repeated for standard luminance visual acuity (Overall model), low luminance visual acuity, and low-luminance deficit in early treatment diabetic retinopathy study (ETDRS) letters. Models bootstrapped 100-fold with resultant regression coefficients (r^2^) and mean absolute error (MAE) are shown.
