# Supplementary material for: Prediction of visual function from automatically quantified optical coherence tomography biomarkers in patients with geographic atrophy using machine learning
Source: Sci Rep. 2022 Sep 16;12:15565. doi: 10.1038/s41598-022-19413-z (PMC9481631; doi:10.1038/s41598-022-19413-z)
Supplement: Supplementary file 2 — Supplementary Table 1. [file 41598_2022_19413_MOESM2_ESM.docx]

|  | R^2^ | MAE  (25% quartile - 75% quartile) |
| --- | --- | --- |
| **Standard visual acuity** |  |  |
| Overall | 0.40 | 12.0 (11.3 - 12.6) |
| FILLY | 0.46 | 10.1 (9.5 - 10.8) |
| MEH | 0.30 | 15.5 (13.8 - 16.5) |
| **Low-luminance visual acuity** |  |  |
| FILLY | 0.25 | 12.1 (11.3 - 12.8) |
| **Low-luminance deficit** |  |  |
| FILLY | 0.20 | 10.3 (9.5 - 11.2) |

**Supplementary Table 1. Feature importances of structure-function correlations at patient-level split.** From our cohort, one eye (randomly selected) per patient was taken forward and structure-function correlation models were repeated for standard luminance visual acuity, low luminance visual acuity, and low-luminance deficit in early treatment diabetic retinopathy study (ETDRS) letters. Model bootstrapped 100-fold with resultant Correlation coefficients (R^2^) and mean absolute error (MAE) shown.
