# Supplementary figures and images for: Prediction of visual function from automatically quantified optical coherence tomography biomarkers in patients with geographic atrophy using machine learning
Source: Sci Rep. 2022 Sep 16;12:15565. doi: 10.1038/s41598-022-19413-z (PMC9481631; doi:10.1038/s41598-022-19413-z)

**A**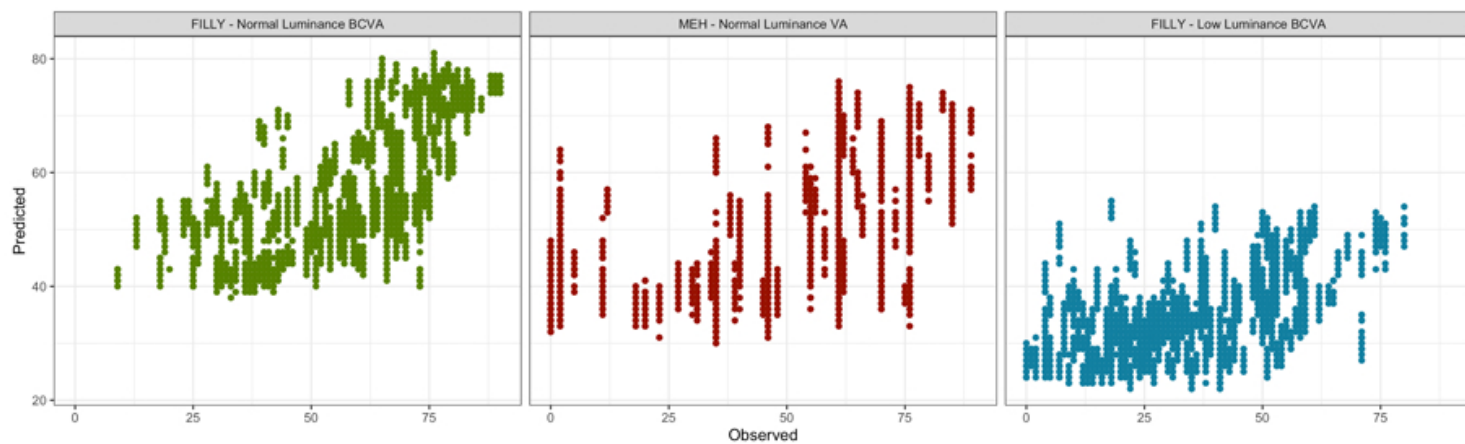**B**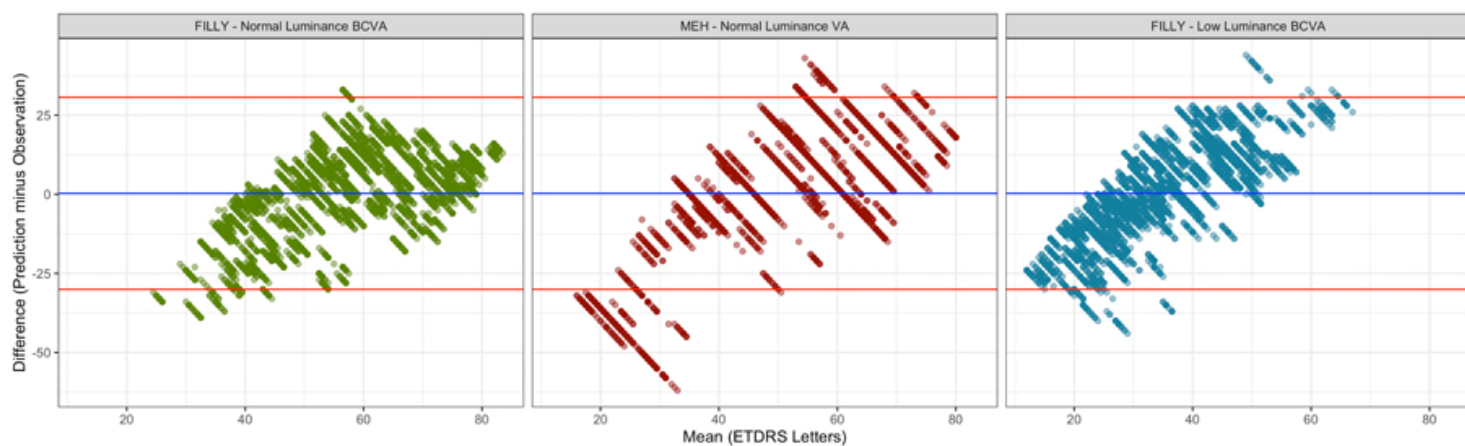

Supplement: Supplementary file 5 — Supplementary Figure 3. [file 41598_2022_19413_MOESM5_ESM.pdf]
